# Identification of Key Biomarkers and Pathways for Maintaining Cognitively Normal Brain Aging Based on Integrated Bioinformatics Analysis

**DOI:** 10.3389/fnagi.2022.833402

**Published:** 2022-03-09

**Authors:** Jinling Xu, Hui Zhou, Guangda Xiang

**Affiliations:** ^1^The First School of Clinical Medicine, Southern Medical University, Guangzhou, China; ^2^Department of Endocrinology, General Hospital of Central Theater Command, Wuhan, China; ^3^Department of General Surgery, The Third Xiangya Hospital, Central South University, Changsha, China

**Keywords:** brain aging, immunity, inflammatory, differentially expressed genes, hub genes

## Abstract

**Background:**

Given the arrival of the aging population has caused a series of social and economic problems, we aimed to explore the key genes underlying cognitively normal brain aging and its potential molecular mechanisms.

**Methods:**

GSE11882 was downloaded from Gene Expression Omnibus (GEO). The data from different brain regions were divided into aged and young groups for analysis. Co-expressed differentially expressed genes (DEGs) were screened. Functional analysis, protein–protein interaction (PPI) network, microRNA (miRNA)-gene, and transcription factor (TF)-gene networks were performed to identify hub genes and related molecular mechanisms. AlzData database was used to elucidate the expression of DEGs and hub genes in the aging brain. Animal studies were conducted to validate the hub genes.

**Results:**

Co-expressed DEGs contained 7 upregulated and 87 downregulated genes. The enrichment analysis indicated DEGs were mainly involved in biological processes and pathways related to immune-inflammatory responses. From the PPI network, 10 hub genes were identified: C1QC, C1QA, C1QB, CD163, FCER1G, VSIG4, CD93, CD14, VWF, and CD44. CD44 and CD93 were the most targeted DEGs in the miRNA-gene network, and TIMP1, HLA-DRA, VWF, and FGF2 were the top four targeted DEGs in the TF-gene network. In AlzData database, the levels of CD44, CD93, and CD163 in patients with Alzheimer’s disease (AD) were significantly increased than those in normal controls. Meanwhile, in the brain tissues of cognitively normal mice, the expression of CD44, CD93, and CD163 in the aged group was significantly lower than those in the young group.

**Conclusion:**

The underlying molecular mechanisms for maintaining healthy brain aging are related to the decline of immune-inflammatory responses. CD44, CD93, and CD 163 are considered as potential biomarkers. This study provides more molecular evidence for maintaining cognitively normal brain aging.

## Introduction

Aging is an inevitable physiological phenomenon manifested by progressive decline of physical function and also increases susceptibility to develop many age-related diseases, such as cardiovascular disease, malignant, and neurodegenerative disorders (North and Sinclair, 2012; de Magalhães, 2013; Hou et al., 2019). Especially, as aging drives cerebral dysfunction and cognitive decline that contribute to both a poor quality of life and short life expectancy, understanding its physiological mechanisms through establishing molecular biomarkers underlying normal brain aging will provide novel insights into anti-aging strategies.

In the process of brain aging, the imbalance of intracellular homeostasis caused by a variety of factors induces cell aging and then affects functional capabilities of the brain, including decrements in learning and memory, processing speed, attention, sensory perception, and motor coordination (Mattson and Arumugam, 2018; Toufektchan and Toledo, 2018). As detailed in recent studies, the cellular and molecular mechanisms of brain aging are complex and mainly include (Li et al., 2016; Kudryashova et al., 2020): (1) dysfunction of mitochondria; (2) accumulation of oxidatively damaged proteins, nucleic acids, and lipids in brain cells; (3) disorders of energy metabolism; (4) impaired “waste disposal” mechanism (autophagosome and proteasome functionality); (5) impaired signal transduction of adaptive stress response; (6) impaired DNA repair; (7) abnormal neural network activity; (8) imbalance of neuronal Ca^2+^ processing; (9) stem cell exhaustion; (10) increased inflammation. Although tremendous advances have been made over recent years, the aging biomarkers that can maintain normal brain aging, as well as the biological processes that cause the progressive loss of healthy physiology are not fully understood. So we focus on healthy brain aging and try to find potential biomarkers and molecular mechanisms for maintaining healthy brain aging.

The rapidly developed high-throughput system has promoted the progression of bioinformatics analysis, which has emerged as a constructive tool to screen out molecular targets related to several diseases. Using this new approach, mRNA gene expression profile GSE11882 from the Gene Expression Omnibus (GEO) database was analyzed to obtain the significant differentially expressed genes (DEGs) between cognitively normal aged brain samples and young brain samples, so as to further explore the key physiological targets and related biological processes of healthy brain aging. Subsequently, the DEGs were subjected to functional and pathway enrichment analysis. Then, network analyses were performed to help us clarify the biological processes and molecular mechanisms. This study aimed to establish a comprehensive network of genes associated with cognitively normal brain aging, enhancing our understanding of the development and protection of brain aging at the molecular biological level.

## Materials and Methods

### Gene Expression Microarray Data and Data Processing

**FIGURE 1:**
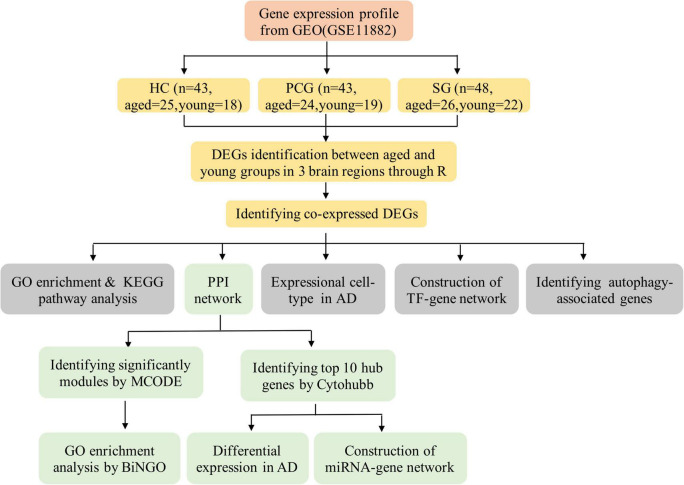
Flowchart of the study.

Data analysis procedures of this study are shown in Figure 1. The original microarray dataset of GSE11882 was downloaded from the National Center of Biotechnology Information-GEO (NCBI-GEO),^1^ which is an international public availability repository. We conducted the search strategy using the following keywords: [“aging” (MeSH Terms) or aging (all fields)] and [“brain” (MeSH terms) or brain (all fields)] and [“*Homo sapiens*” (organism) and “expression profiling by array” (filter)]. GSE11882 dataset is a cognitively normal subjects gene expression profile (age 20–99 years) (Berchtold et al., 2008) based on the GPL570 platform. It includes 173 postmortem brain tissue from 4 brain regions: 43 hippocampus (HC), 39 entorhinal cortex (EC), 48 superior frontal gyrus (SG), and 43 postcentral gyrus (PCG). In this study, we reextracted data on the basis of different brain regions. In each brain region, the samples were divided into the aged group (≥ 60 years old) and the young group (< 60 years old). In each brain region, the numbers of aged individuals and young individuals are shown in Figure 1. Then, we analyzed differential expression between aged and young groups in different brain regions. The annotation files for GPL570 and the CEL files of GSE11882 were also downloaded from GEO. Raw CEL data were preprocessed into expression values with Affy V1.68.0 package of R software (version 4.0.3), and then the gc*rma* V2.62.0 package was applied to correct the background and normalize the gene expression profiles. Quality control analysis was performed based on ArrayQualityMetrics V3.46.0 package.

### Identification of Differentially Expressed Genes

To identify DEGs, normalized log-expression data in the aged and young groups were analyzed by limma (linear models for microarray data) V3.46.0 package in the R/Bioconductor platform. Benjamini–Hochberg’s approach was used to adjust the false discovery rate (FDR) and genes with the adjusted *P*-value < 0.05 and | Log2 fold-change (log2FC)| > 1 were selected as DEGs. All of the DEGs are visualized with the volcano plot generated by R software ggplot2 V3.3.2 package; the significant genes with | log2FC| > 2 are shown in the volcano plot. Then, the heatmap for the 50 most significant DEGs from each dataset was plotted by using R software Pheatmap V1.0.12 package. The visualization of overlapping DEGs was analyzed by the means of VennDiagram function from the VennDiagram V1.6.20 package in R.

### Gene Ontology and Kyoto Encyclopedia of Genes and Genomes Pathway Enrichment Analysis

The Gene Ontology (GO) analysis has merged as a useful and widespread approach to perform functional enrichment research of large-scale genomic data. The GO terms (biological process, cellular component, and molecular function) as well as the Kyoto Encyclopedia of Genes and Genomes (KEGG) pathways enrichment analysis of the co-expression DEGs between the aged and young groups were conducted and visualized based on the clusterProfiler version 3.18.0 package in R software (significant as *P* < 0.05 and a *q*-value < 0.05) and the Metascape (significant as *P* < 0.01, minimum overlap = 3, and minimum enrichment = 1.5), a user-friendly, publicly available online tool for gene annotation integration. In addition, the GO and KEGG pathway analyses of DEGs were also performed through DAVID 6.8 online tool,^2^ with the criteria as follows: Benjamini-adjusted *P* < 0.05 and an enriched gene count > 5.

### Protein–Protein Interaction Network Construction and Analysis

Protein–protein interaction information of the DEGs was acquired using the search tool for the retrieval of interacting genes (STRING) database (V11.0),^3^ an open-source online database providing prediction of protein functional associations. The standard of significant interaction was set as a combined score ≥ 0.4. Then, Cytoscape software (version 3.8.2)^4^ was employed to construct and visualize the PPI network according to PPI information. Moreover, we successively performed module analysis and GO analysis to illuminate the biological processes that the module genes were significantly enriched by two Cytoscape plug-ins, namely, molecular complex detection (MCODE, V2.0.0) and the biological network GO tool (BiNGO, V3.0.4), respectively. In order to determine the hub genes contained in the PPI network, cytohubba V0.1 plug-in was applied to screen out essential nodes with the maximal clique centrality (MCC) method. All the parameters were set by defaults.

### Construction of Cell-Type-Gene Network and Verification of Hub Genes Expression in Alzheimer’s Disease

AlzData database^5^ contains high throughput data collection of Alzheimer’s disease (AD), including single-cell expression and differential expression. In this study, the DEGs lists were put into AlzData database to obtain each gene-related cell type, providing the information for the construction of cell-type-gene network by Cytoscape software. Next, we searched CD44, CD93, and CD163 to verify the differential expression in AD.

### Establishment of MicroRNA-Gene and Transcription Factor-Gene Networks

MicroRNA (miRNA) or transcription factor (TF) regulates the posttranscriptional modification of gene expression under a disease condition *via* interaction with target genes. To further clarify the regulation of DEGs expression, we used TargetScan,^6^ ENCORI,^7^ miRDB,^8^ and TRRUST database^9^ to predict miRNA and TFs of DEGs and then both the miRNA-gene and TF-gene networks were established by Cytoscape software.

### Selection of Autophagy-Associated Genes

The genes list related to autophagy were retrieved from the GeneCards website^10^ using the search term “autophagy.” A relevance score (rang 0–100) presents the strength of the correlation between genes and autophagy activity. Candidate genes were defined as association score > 3 and ranked based on their scores. Autophagy-associated genes were screened through the intersection of DEGs and candidate genes. The correlation matrix of autophagy-associated genes was calculated and visualized by corrplot V0.84 package in R.

### Identification and Screening of Cognitively Normal Mice

A 2-month-old male C57BL/6J mice (*n* = 6) and 15-month-old male C57BL/6J mice (*n* = 20) were performed Morris water maze training to evaluate the cognitive function. The animals were given 15 trials over 5 consecutive days with the platform submerged 2 cm below the surface of the water (three trials per day; 20–30 min intertrial interval). During the training, the mice were put into the pool facing the pool wall and the time from entering the water to finding the underwater hidden platform and standing on it was recorded, which was defined as the escape latency. If the mice could not find the platform 60 s after entering the water, the operator would guide them to the platform, stay for 10 s, and set the latency to 60 s. The average of escape latency of the day was the test results. The aged mice were considered normal cognition if their mean latency score was ≤ 0.5 SD from the mean of the young controls, while the mean latency score > 3 SD was identified the impaired cognition. Mice with impaired cognition were eliminated. After screening, the mice were anesthetized with pentobarbital sodium (60 mg/kg) and the hippocampal tissues were isolated. The C57BL/6J mice were purchased from Vital River Laboratory Animal Technology Corporation Ltd. (Beijing, China). Animal procedures conformed to the National Institutes of Health Guidelines for the Use of Laboratory Animals and were approved by the Animal Ethics Committee of General Hospital of Central Theater Command.

### Quantitative PCR

The expression of CD44, CD93, and CD163 was measured by quantitative PCR (qPCR). Total RNA was extracted from hippocampal tissues using Trizol reagent (Sigma, St. Louis, Missouri, United States) according to the manufacturer’s instructions. The mRNA was reverse transcribed to cDNA using RevertAid First Strand cDNA Synthesis Kit and SYBR Select Master Mix Kit was then performed for qPCR. Relative changes in mRNA levels among groups were determined with 2^–Δ^ ^Δ^ *^Ct^* method. The thermal cycling conditions for RT-PCR were 95°C for 60 s, followed by 40 cycles of 95°C for 10 s, 60°C for 5 s, and 72°C for 10 s. GAPDH was used as the housekeeping gene to normalize the expressions of the target genes. The primers used for amplification were as follows: CD44: Forward Primer (5′-3′) AGAAAAATGGCCGCTACAGTATC, Reverse Prime (5′-3′) TGCATGTTTCAAAACCCTTGC; CD93: Forward Primer (5′-3′) GCCATCTCAACTGGTTTGTTCC, Reverse Primer (5′-3′) ACTCTTCACGGTGGCAAGATT; CD163: Forward Primer (5′-3′) ATGGGTGGACACAGAATGGTT, Reverse Primer (5′-3′) CAGGAGCGTTAGTGACAGCAG; GAPDH: Forward Primer (5′-3′) TGGCCTTCCGTGTTCCTAC, Reverse Prime (5′-3′) GAGTTGCTGTTGAAGTCGCA.

### Western Blot

Supernatants of hippocampal tissues homogenates were electrophoresed and then transferred to a nitrocellulose membrane. The membranes were blocked for 1 h at room temperature with Blotto-Tween and incubated with primary antibody, CD44 (1:600 dilution), CD93 (1:600 dilution), and CD163 (1:600 dilution) at 4°C (all purchased from Boster Bioengineering Corporation, Wuhan, China). Bound antibody was detected with horseradish peroxidase-labeled secondary antibodies (1:5,000) (Boster Bioengineering Corporation, Wuhan, China). Blots were quantified with ImageJ software.

## Results

### Identification of Differentially Expressed Genes

**FIGURE 2:**
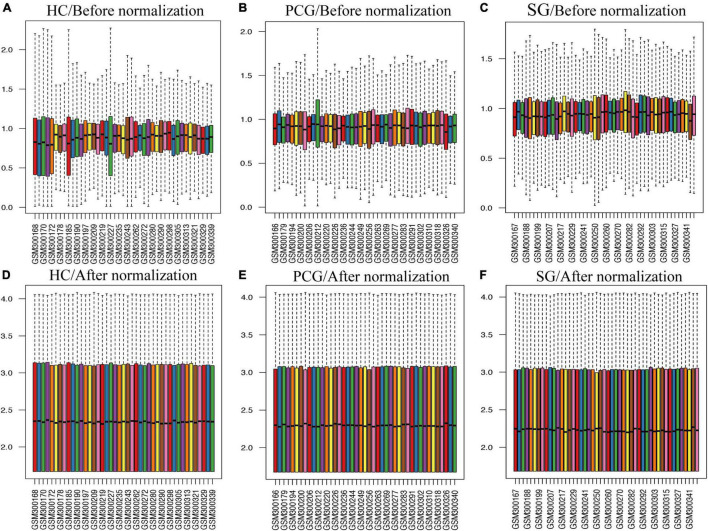
Box plots of the gene expression data between aged and young groups in HC, SG, and PCG before and after normalization. (A–C) Before normalization; (D–F) after normalization. The *x*-axis label represents the sample symbol and the *y*-axis label represents the gene expression values. The black line in the box plot represents the median value of gene expression. HC, hippocampus; PCG, postcentral gyrus; SG, superior frontal gyrus.

**FIGURE 3:**
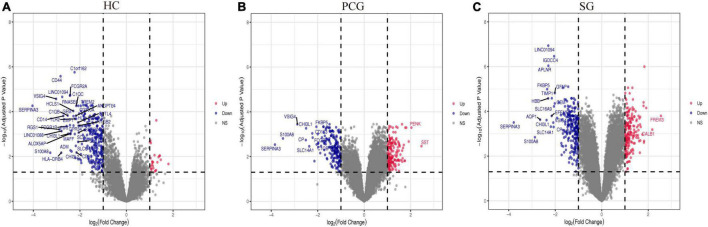
Volcano plots of all the differentially expressed genes (DEGs) between aged and young groups in HC (A), PCG (B), and SG (C). Nodes in red represent up-regulated genes, nodes in blue represent down-regulated genes, and gray dots represent no significantly changed genes. The differences are set as | log FC| > 1. The genes with | log FC| > 2 were marked with gene names. HC, hippocampus; PCG, postcentral gyrus; SG, superior frontal gyrus.

**FIGURE 4:**
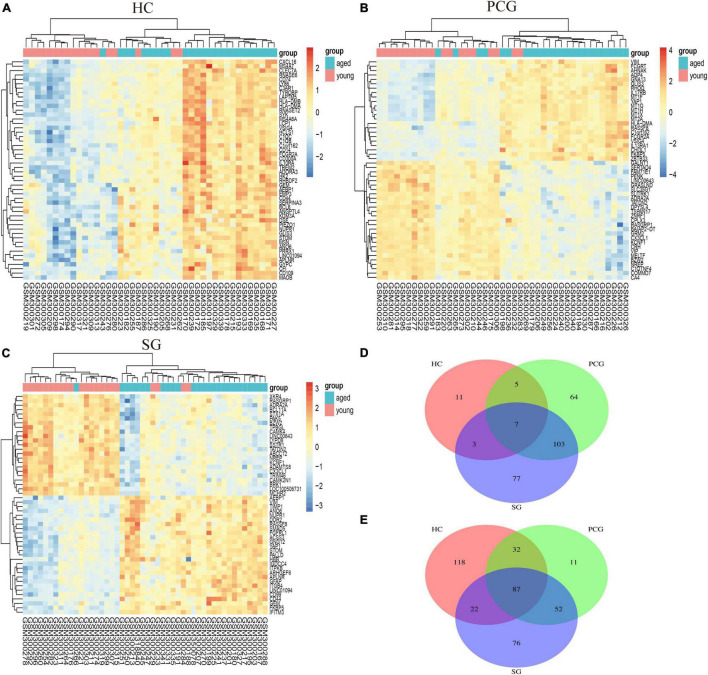
Heatmap and Venn diagram of DEGs. (A–C) Heatmaps of DEGs between aged and young groups in HC, SG, and PCG. Legend on the top right indicates the log fold change of the genes. The horizontal axis represents each sample, and the vertical axis represents each gene. Blue and red colors represent low and high expression values, respectively. (D,E) Venn diagrams of upregulated (D) and downregulated (E) DEGs in the datasets of HC, SG, and PCG. The intersection in blue represents the DEGs that are common among the three datasets. HC, hippocampus; PCG; postcentral gyrus; SG, superior frontal gyrus.

As shown in Figure 2, the mean values of gene expression for each sample in different brain regions were fundamentally the same after normalization (Figure 2), indicating that the source of sample data was reliable. After comparison of the aged and young groups in HC, PCG, and SG, 284 genes (25 upregulated and 259 downregulated genes), 359 genes (178 upregulated and 181 downregulated genes), and 426 genes (190 upregulated and 236 downregulated genes) were identified as DEGs respectively. The distribution of these DEGs was presented in the volcano plots and the heatmaps (Figures 3, 4A–C). Since no DEGs were found in EC tissues, we excluded the data about EC tissues, and the following analysis mainly focused on HC, PCG, and SG tissues. To determine the shared DEGs that contribute to cognitively intact brain aging, Venn analysis was used among HC, PCG, and SG. As shown in Figures 4D,E and Table 1, seven upregulated co-expressed genes and 87 downregulated co-expressed genes were identified in healthy brain aging.

**TABLE 1:** Differentially expressed genes of healthy brain aging.

DEGs	Gene symbol
Up regulated genes	DGKI/ATP2B2/PCDHAC2/PNOC/CACNB4/CRH/CNTNAP5
Down regulated genes	C1orf162/CD44/LINC01094/VSIG4/APLNR/SERPINA3/ STOM/CD109/C1QC/HCLS1/GLIS3/AEBP1/ANO6/C1QB/ ANGPTL4/NUPR1/CD14/GEM/HLADRA/RFX4/SLC7A2/IFIT M3/CP/IFITM2/GFAP/CAPG/BAG3/ID3/TMEM176B/RASL12/ ITGB4/SYTL4/IL13RA1/S1PR3/GPR34/FCER1G/C1QA/FCG R1B/TIMP1/CLEC2B/DDIT4/ITPKB/CHI3L1/ACSL5/TGIF1/TM EM176A/MT1X/MT1H/MGST1/VWF/LRRC32/SRPX/IGDCC4/ YBX3/S100A11/DDIT4L/MT1G/SLC14A1/FKBP5/SLCO4A1/ MT1M/FAM189A2/PLSCR4/CFH/TGFBR3/CD93/HLADRB4/ S100A8/ANGPT2/VAMP8/CD163/YAP1/PHYHD1/FGF2/AQ P1/IL1R1/HBB/ANXA2/FCGBP/IL1RL1/HSPB1/HIF3A/MYOF/ AHNAK/TUBB6/SLC16A9/HLA-DMA

*DEGs, differentially expressed genes.*

### Gene Ontology and the Kyoto Encyclopedia of Genes and Genomes Pathway Functional Enrichment Analysis of Differentially Expressed Genes

**FIGURE 5:**
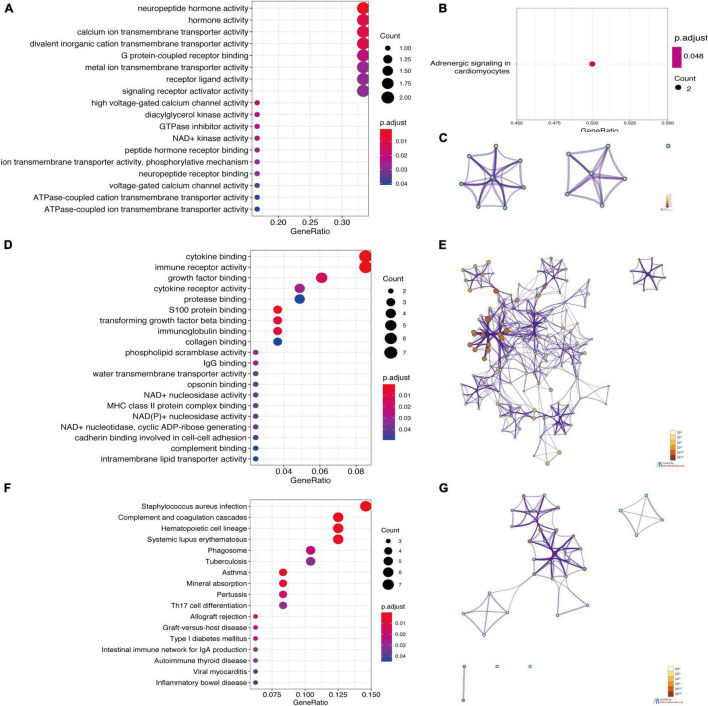
Enrichment analysis of DEGs based on clusterProfiler packages and Metascape. (A) The GO enrichment items of up-regulated DEGs. (B) The KEGG pathway enrichment results of upregulated DEGs. (C) Network of the GO enriched terms of the upregulated DEGs. (D) The GO enrichment items of downregulated DEGs. (E) Network of the GO enriched terms of downregulated DEGs. (F) The KEGG pathway enrichment results of downregulated DEGs. (G) Network of the KEGG pathway enrichment results of the downregulated DEGs. (A,B,D,F) were analyzed by clusterProfiler packages. The *x*-axis label represents the gene ratio, and the *y*-axis label represents GO terms. The size of circle represents gene count. Different color of circles represents different adjusted *p*-value. (C,E,G) were analyzed by Metascape. The enriched terms are colored by *p*-value, where terms containing more genes tend to have a more significant *p*-value. The most statistically significant term within a cluster was chosen as the one representing the cluster.

The results showed that the top 10 GO enrichment items of upregulated genes were the activity of neuropeptide hormone, hormone, calcium ion transmembrane transporter, divalent inorganic cation transmembrane transporter, high voltage-gated calcium channel, diacylglycerol kinase, GTPase inhibitor and NAD^+^ kinase, and binding of G protein-coupled receptor and peptide hormone receptor (Figures 5A,C). The result of the KEGG pathway enrichment with upregulated DEGs was enriched in adrenergic signaling pathway in cardiomyocytes (Figure 5B). Then, the GO terms of downregulated DEGs were significantly enriched in binding of cytokine, growth factor, protease, S100 protein, transforming growth factor-β (TGF-β), immunoglobulin and collagen, and the activity of immune receptor, cytokine receptor and phospholipid scramblase (Figures 5D,E). Regarding the KEGG pathway, downregulated genes were mainly involved in complement and coagulation cascades, *Staphylococcus aureus* infection, hematopoietic cell lineage, asthma, systemic lupus erythematosus, mineral absorption, pertussis, allograft rejection, graft-vs.-host disease, and phagosome (Figures 5F,G). To provide another option for significant enrichment results, the results of DAVID are given in Supplementary Table 1.

### Protein–Protein Interaction Network Analysis of Differentially Expressed Genes

**FIGURE 6:**
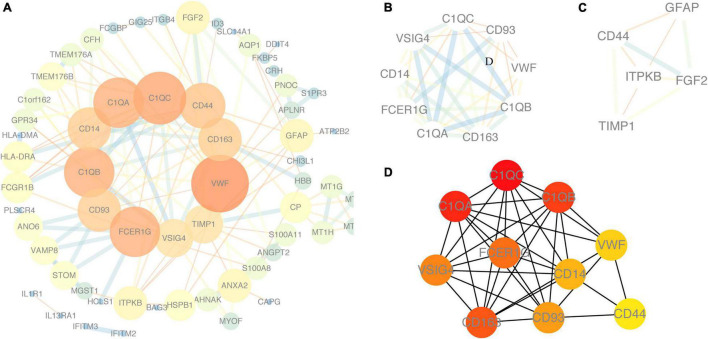
Protein–protein interaction (PPI) network. **(A)** PPI network of DEGs using NetworkAnalyzer plugin. **(B,C)** Module 1 and module 2, the most significant modules were obtained from the PPI network using MCODE plugin. **(D)** Top ten hub genes from the PPI network analyzed by Cytohubb plugin. Red color represents a higher degree, and yellow color represents a lower degree.

Using the STRING online database and Cytoscape software, the PPI network of 94 DEGs consisted of 102 nodes and 201 edges, accompanied with average node degree 3.94 and average local clustering coefficient 0.457 (Figure 6A). The nodes correspond to genes and the edges between the two nodes represented co-expression links. As shown in Figures 6B,C, the two most significant PPI modules were comprised of 9 nodes and 5 nodes, respectively, and the contained genes were all downregulated DEGs. Subsequently, the enriched GO terms of the two modules were analyzed. The 5 most significant GO terms, which are given in Tables 2, 3, were mainly involved in the regulation of inflammatory response, the positive regulation of signaling process, and ureteric bud morphogenesis. Furthermore, the top 10 hub genes were C1QC, C1QA, C1QB, CD163, FCER1G, VSIG4, CD93, CD14, VWF, and CD44 (Figure 6D), which were all downregulated genes, implying that they might play essential roles in the biological process of healthy brain aging (Table 4).

**TABLE 2:** The top five significantly enriched GO terms in module 1.

GO-ID	*p*-value	corr *p*-value	x	n	X	N	Description
2,526	3.47E-10	9.99E-08	5	89	9	17,790	Acute inflammatory response
9,611	7.91E-10	1.14E-07	7	541	9	17,790	Response to wounding
6,954	2.36E-09	1.75E-07	6	315	9	17,790	Inflammatory response
6,956	2.74E-09	1.75E-07	4	40	9	17,790	Complement activation
2,541	3.03E-09	1.75E-07	4	41	9	17,790	Activation of plasma proteins involved in acute inflammatory response

*GO, gene ontology.*

**TABLE 3:** The top 5 significantly enriched GO terms in module 2.

GO-ID	*p*-value	corr *p*-value	x	n	X	N	Description
23,056	3.81E-05	4.05E-03	3	281	5	17,790	Positive regulation of signaling process
9,967	3.61E-05	4.05E-03	3	276	5	17,790	Positive regulation of signal transduction
70,374	3.33E-05	4.05E-03	2	33	5	17,790	Positive regulation of ERK1 and ERK2 cascade
60,675	2.74E-05	4.05E-03	2	30	5	17,790	Ureteric bud morphogenesis
1,658	2.38E-05	4.05E-03	2	28	5	17,790	Branching involved in ureteric bud morphogenesis

*GO, gene ontology.*

**TABLE 4:** Detail information of 10 hub genes in the PPI network.

Gene symbol	Full name	Involved function	Score	Degree
C1QC/C1QA/C1QB	Complement C1q C Chain	Classical complement cascade; innate immune response	1,693/1,692/1,686	15/14/14
CD163	CD163 Molecule	Scavenger receptor activity	1,586	11
FCER1G	Fc Fragment Of IgE Receptor Ig	Transmembrane signaling receptor activity; IgE binding	1,515	14
VSIG4	V-Set And Immunoglobulin Domain Containing 4	Innate and adaptive immunity; immunosuppressive and anti-inflammatory	1,465	9
CD93	CD93 Molecule	Signal transduction; immune functions	866	11
CD14	CD14 Molecule	Immunity and inflammation functions	796	11
VWF	Von Willebrand Factor	Coagulation functions	212	17
CD44	CD44 Molecule	Inflammation related functions; cell surface receptor for hyaluronan	60	12

*PPI, protein–protein interaction.*

### Construction of miRNA-Gene and the Transcription Factor-Gene Networks

**FIGURE 7:**
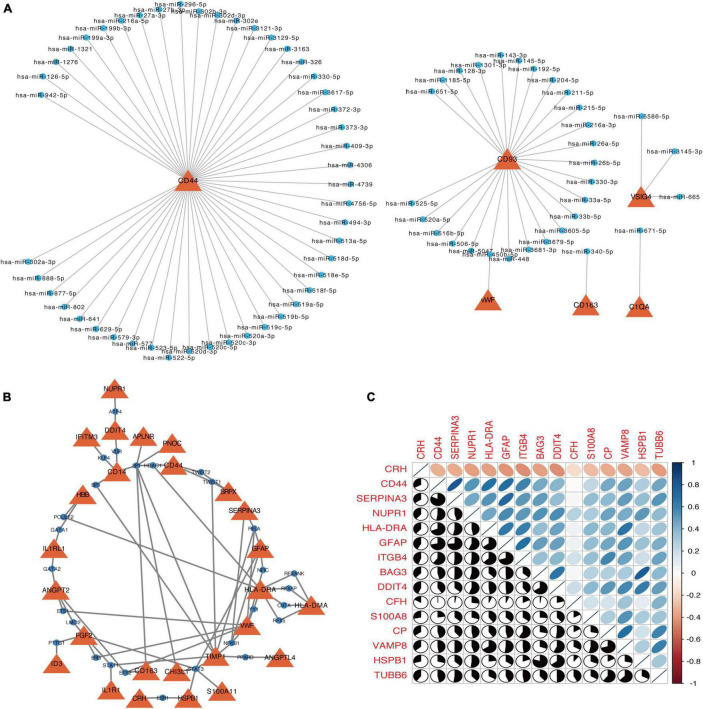
The networks of target gene-miRNA **(A)** and target gene-TF **(B)**. The red triangles are the genes, and blue circle nodes are the miRNAs or TF. **(C)** Correlation matrix of autophagy-related genes in DEGs. Orange indicates a negative correlation and blue indicates a positive correlation.

From miRNA-gene regulatory network, the most potential targeted DEGs for miRNAs were CD44 that was regulated by 47 miRNAs, including hsa-miR-126-5p, hsa-miR-1276, hsa-miR-1321 and so on (Figure 7A). Moreover, CD93 was regulated by 26 miRNAs (Figure 7A). Next, we furtherly predicted potential TFs for DEGs. The top four targeted DEGs for TFs were TIMP1, HLA-DRA, VWF, and FGF2, which were modulated by 8 TFs, 7 TFs, 7 TFs, and 5 TFs, respectively. Among the 28 TFs, TF SP1 regulated the largest number of DEGs (7 genes) (Figure 7B).

### Selection of Autophagy-Related Genes in Differentially Expressed Genes

In DEGs, a total of 15 autophagy-associated genes were identified, including CRH, CD44, SERPINA3, NUPR1, HLA-DRA, GFAP, ITGB4, BAG3, DDIT4, CFH, S100A8, CP, VAMP8, HSPB1, and TUBB6. Figure 7C shows the correlation among these autophagy-associated genes, and six combinations [SERPINA3 and CD44 (*r* = 0.82), HSPB1 and BAG3 (*r* = 0.80), GFAP and SERPINA3 (*r* = 0.73), GFAP and CD44 (*r* = 0.72), GFAP and ITGB4 (*r* = 0.71), and CP and VAMP8 (*r* = 0.71)] had high degrees of interaction connectivity.

### Single-Cell Expression of Differentially Expressed Genes and Differential Expression of Hub Genes in Alzheimer’s Disease

**FIGURE 8:**
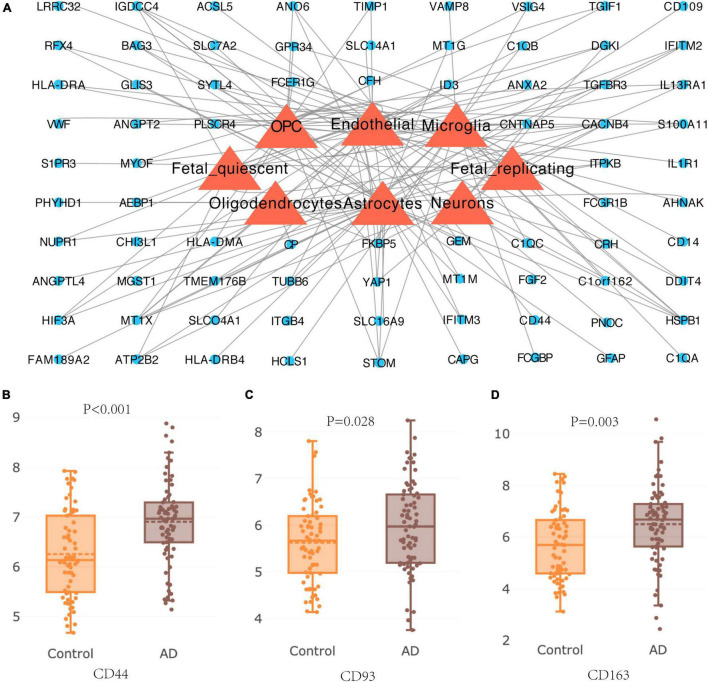
Cell-type expression of DEGs and differential expression of hub genes in Alzheimer’s disease. **(A)** Cell type gene of DEGs in brain tissue of patients with Alzheimer’s disease. **(B–D)** Differential expression of hub genes CD44, CD93, and CD163 in hippocampus of patients with Alzheimer’s disease.

The lists of DEGs were introduced into AlzData online database to elucidate the relationship between 94 DEGs and cell types in the brain. A cell-type-gene network was constructed on the basis of the above information (Figure 8A), and we found 39 genes were expressed in astrocytes, 36 genes were expressed in endothelial, 25 genes were expressed in microglia, 24 genes were expressed in oligodendrocyte precursor, and 9 genes were expressed in neurons, indicating effects of aging on these cells (Figure 8A). AlzData database was further employed to obtain the differential expression of hub genes in HC tissue, revealing expression of CD44, CD93, and CD163 in patients with AD were significantly increased than that in normal controls (Figures 8B–D).

### Expression of CD44, CD93, and CD 163 in Cognitively Normal Mice

**FIGURE 9:**
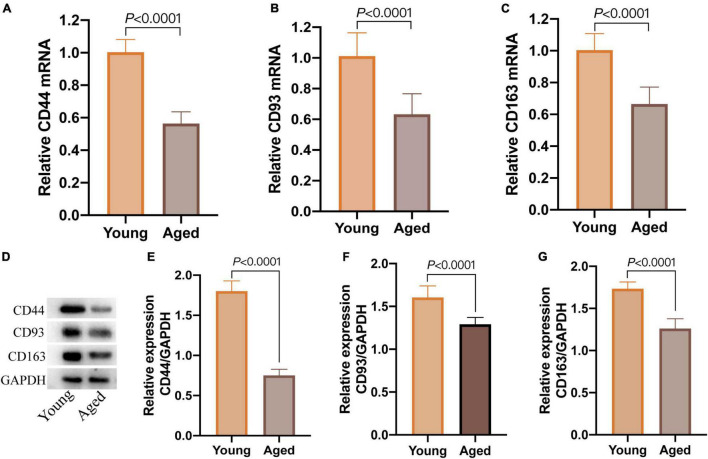
The expression of CD44, CD93, and CD163 in hippocampal tissue of cognitively normal aged and young mice. **(A–C)** The relative expression of CD44, CD93, and CD163 mRNA detected by qPCR; **(D)** western blot images of CD44, CD93, and CD163; **(E–G)** the levels of CD44, CD93, and CD163 protein detected by western blot.

After screening by Morris water maze training, 8 cognitively normal aged mice were chosen (aged group, *n* = 8), and 6 cognitively normal young mice were considered as a control group (young group, *n* = 6). qPCR revealed that the relative expression of CD44, CD93, and CD163 mRNA in the aged group was significantly lower than those in the young group (Figures 9A–C); simultaneously, the protein relative expression levels in the aged group were also significantly down-regulated (Figures 9D–G). These results showed that the expression of CD44, CD93, and CD163 was downregulated in cognitively normal aged mice, which were consistent with the dataset of GSE11882.

## Discussion

Population aging has become a major public health problem in the world. Targeted strategies to brain aging based on the pathophysiology, compared with treating each age-associated neurological impairments separately, may have better therapeutic effects. However, the fundamental mechanisms that drive healthy brain aging have not been fully clarified. In this perspective, we aimed to discover the key genes and pathways that contribute to human healthy brain aging by bioinformatics analyses.

Based on GSE11882, we extracted the expression matrix of aged brain samples and young brain samples in HC, PCG, and SG, respectively. A total of 94 common DEGs were identified among the three different brain regions, composed of 87 downregulated genes and 7 upregulated genes. Functional enrichment analysis showed that the significant GO terms enriched in upregulated genes were mainly neuropeptide hormone activity, peptide hormone receptor binding, regulation of calcium ion transportation, GTPase inhibitor, and NAD^+^ kinase. Consistent with the previous evidence, neuropeptide hormones, such as neuropeptide Y (NPY), brain-derived neurotrophic factor (BDNF), somatostatin (SST), and corticotropin releasing hormone (CRH), are involved in brain aging and considered as positive factors against brain aging (Sadow and Rubin, 1992; Viollet et al., 2008; Botelho and Cavadas, 2015; Molinari et al., 2020). Specifically, NPY contributes to and counteracts aging-related hallmarks (Botelho and Cavadas, 2015), such as disturbed proteostasis, stem cell exhaustion, alteration of intercellular communication, deregulated nutrient sensing, cellular senescence, and mitochondrial dysfunction. Moreover, cellular calcium dyshomeostasis, as an accepted hallmark of aging, contributes to not only aging initiation, but also progression (Green, 2009). Indeed, age-related changes in various calcium channels, receptors, and pumps have been shown to cause changes during the process of the amyloid precursor protein (Oulès et al., 2012). NAD+ kinases are an essential and ubiquitous enzyme involved in the tight regulation of NAD/NAD phosphate levels in many metabolic pathways. Consistently, cellular NAD levels decrease in the process of chronological aging (Oulès et al., 2012). NAD decline seemingly plays a role in the development of age-related diseases, suggesting that NAD replacement treatment may open a new avenue for age-related cerebral disorders.

Significant GO terms of downregulated genes were commonly associated with immune and inflammatory responses. At present, abundant evidences have confirmed that the various senescence-associated secretory phenotype (SASP) components, such as inflammatory molecules (such as cytokines and chemokines), growth factors and regulators (such as TGF-β, insulin-like growth factor-binding proteins), extracellular proteinases, and their inhibitors, are among candidate biomarkers of aging. These descriptions are in line with the results of our GO analysis, which show that cytokine binding, growth factor binding, TGF-β binding, protease binding, and cytokine receptor activity are the significant GO terms. Besides, downregulated genes were also enriched in immunoglobulin binding, IgG binding, and immune receptor activity. These studies have shown that altered immune responses within the brain and in the periphery affect the function of brain aging leading to impaired neurogenesis and cognition (Shaw et al., 2013; Jin et al., 2021). Additionally, S100 protein, another significant GO term, is observed to regulate several signaling pathways and levels of cytokines associated with AD (Cristóvão and Gomes, 2019). Based on these results, it is speculated that the declined cellular immune and inflammatory system might play a pivotal role in keeping healthy brain aging.

The KEGG enrichment analysis of downregulated DEGs determined that these DEGs were mapped in the complement and coagulation cascades, and phagosome, both of which were consistent with the previous demonstration that aging has cross-talking with immune inflammation and autophagy (Tzoran et al., 2018; Xu et al., 2020). Corresponding to the enriched KEGG pathway of mineral absorption, selenium, zinc, iron, and copper absorption might be inversely correlated with the prevalence of low cognitive performance in elderly (Steinbrenner and Klotz, 2020).

To screen the key genes that might keep healthy brain aging, we carried out PPI network analysis and determined 10 hub genes, including C1QC, C1QA, C1QB, CD163, FCER1G, VSIG4, CD93, CD14, VWF, and CD44. All of these hub genes were downregulated in the elderly, which exerted a considerable impact on the initiation and development of brain aging from different aspects. C1QC, C1QA, and C1QB are three similar but distinct subunits of C1Q, which is the main protein of classical complement cascade (Sellar et al., 1991). Several lines of evidence show that C1Q has been implicated in aging. It has been reported that C1Q deficiency does not show any functional abnormalities of HC in physiological conditions, while its activation in the brain may contribute to the progression of age-related cognitive dysfunction (Beglopoulos et al., 2004; Cho, 2019). According to a previous study, non−classical CD163+ monocytes are significantly age-related higher whereas classical CD163^+^ significantly age-related lower in older than younger healthy control individuals, suggesting circulating CD163 monocytes could be a novel predictor to monitor healthy aging (Costantini et al., 2018). By now, FCER1G has been identified as a possible microglial biomarker associated with both human aging and neurodegeneration (Mukherjee et al., 2019). It is indicated that, VSIG4, considered as an immune checkpoint protein and complement receptor, is a novel biomarker of chronological and biological aging (Hall et al., 2020). CD93, which is known for its immune functions, is confirmed to be a negative regulator in astrogenesis and participates in the regulation of central nervous system inflammation following injury (Griffiths et al., 2018; Liang et al., 2020). Regarding CD14, it is an inflammatory marker positively related to brain atrophy, cognitive impairment, and incident dementia (Pase et al., 2020). The plasma concentration of VWF increases with progressing age in healthy humans, while VWF cleaving proteases levels are low in centenarians (Mari et al., 2008). The data show the high level of the VWF is compatible with health and longevity. For CD 44, Pinner et al. (2017) report that it may contribute to AD pathology through cell adhesion, and migration of immune cells, astrocytes, and microglia. Consistently, modules analysis of the PPI network further confirmed that GO terms mainly enrich in immunity and inflammation. Therefore, the above results speculate the viewpoint that these ten hub genes might play critical roles in the molecular level of declined activity of age-related immune and inflammatory system in the brain, providing us some potential therapies for anti-brain aging by targeting these hub genes.

Subsequently, miRNA-gene and TF-gene networks were constructed. The most targeted DEGs in miRNA-gene network were CD44 and CD93, and the top four targeted DEGs in TF-gene network were TIMP1, HLA-DRA, VWF, and FGF2. A growing number of studies have unveiled that TIMP1, HLA-DRA, and FGF2 play neuroprotective roles against brain aging through some mechanisms, such as regulation of immune activity, maintain the integrity of junctional proteins and trans-endothelial tightness of human brain microvessel endothelial cells, and augment of neuron survival, neurogenesis and nerve repair (Yin et al., 2017; Tang et al., 2020). SP1, which controlled most DEGs, plays an essential role in neuroinflammation of aged HC and might be related to cognitive development during brain aging (Gavilán et al., 2009; Gaur and Prasad, 2014). The roles of CD44, CD 93, and VWF in the human brain have been discussed previously.

Autophagy has a prominent role in the life-span regulation of many model organisms. Autophagy defects can accelerate the aging process, whereas activation of autophagy might have potent anti-aging effects (Madeo et al., 2010). In the analysis related to autophagy genes, GFAP and SERPINA3, CD44, ITGB4 may be synergistic in the preservation of healthy brain aging. Further research regarding the relationship between these autophagy-associated genes with aging still needs to be explored.

To further confirm the correlation between genes and brain aging, the expression data of DEGs were acquired from AlzData online database. DEGs were mainly expressed in astrocytes, endothelial cells, and microglia, which play crucial roles in learning and memory. Importantly, expressions of CD44, CD93, and CD163 were found to be higher in HC of patients with AD as compared to the normal controls, while they are downregulated in healthy brain aging, indicating that CD44, CD93, and CD163 might be the key therapeutic targets for healthy brain aging. Meanwhile, the cognitively normal aged mice were screened, and it was found that the expression of CD44, CD93, and CD163 was down-regulated in the HC tissue of the cognitively normal aged mice.

Nonetheless, this study has some limitations that need to be addressed. On one hand, due to the lack of data about the different cell-type expression of genes in normal brain aging, we used the AlzData database to explore the single-cell expression of DEGs and differential expression of hub genes. Although this may not be optimal, it is sufficient to validate the cellular localization of DEGs and differential expression of hub genes in brain aging. Furthermore, the animal experiment also validated the expression of hub genes. Therefore, more clinical and animal-based studies are required in order to better elucidate these associations. On the other hand, because GSE11882 did not provide complete clinical demographic characteristics, we cannot make a related analysis to explore whether there are underlying factors that biased our results. We will further improve this in future clinical studies.

## Conclusion

In summary, this study uncovered 94 DEGs and 10 hub genes involved in cognitively intact brain aging. It was also found that the important pathways were associated with immunity and inflammation. Therefore, it is speculated that declined immune-inflammatory response may be the key mechanism for maintaining healthy brain aging. CD44, CD93, and CD 163 are considered as the potential biomarkers. In addition, we further analyzed the target genes for miRNA/TFs, screened out autophagy-associated genes, which provided more useful information for us to understand the regulation of brain function in normal cognitive elderly. In the future, more studies are urgently warranted to better clarify the biological functions of these genes and pathways in cognitively normal brain aging.

## Data Availability Statement

The datasets presented in this study can be found in online repositories. The names of the repository/repositories and accession number(s) can be found below in the article/Supplementary Material.

## Ethics Statement

The animal study was reviewed and approved by the Animal Ethics Committee of General Hospital of Central Theater Command.

## Author Contributions

JX and HZ were responsible for data acquisition and analysis. JX drafted the manuscript. GX designed the study and revised the article. All the authors approved the manuscript for submission.

## Conflict of Interest

The authors declare that the research was conducted in the absence of any commercial or financial relationships that could be construed as a potential conflict of interest.

## Publisher’s Note

All claims expressed in this article are solely those of the authors and do not necessarily represent those of their affiliated organizations, or those of the publisher, the editors and the reviewers. Any product that may be evaluated in this article, or claim that may be made by its manufacturer, is not guaranteed or endorsed by the publisher.

## Footnotes

1
http://www.ncbi.nlm.nih.gov/geo

2
https://david.ncifcrf.gov/tools.jsp

3
https://cn.string-db.org

4
http://www.cytoscape.org/

5
http://www.alzdata.org/

6
http://www.targetscan.org/mamm_31/

7
http://starbase.sysu.edu.cn/index.php

8
http://mirdb.org

9
https://www.grnpedia.org/trrust/

10
https://www.genecards.org/
