# Supplementary material for: Identification of Key Biomarkers and Pathways for Maintaining Cognitively Normal Brain Aging Based on Integrated Bioinformatics Analysis
Source: Front Aging Neurosci. 2022 Mar 9;14:833402. doi: 10.3389/fnagi.2022.833402 (PMC8959911; doi:10.3389/fnagi.2022.833402)
Supplement: Supplementary file 1 [file Table_1.DOCX]

**Table s1 The top five GO terms and KEGG pathways in enrichment analyses of DEGs**

| Category Term | Description | Count | *P* Value |
| --- | --- | --- | --- |
| *Up-regulated genes* | |  |  |
| BP GO:0006955 | Immune response | 11 | 3.38E-05 |
| BP GO:0045926 | Negative regulation of growth | 4 | 9.91E-05 |
| BP GO:0071294 | Cellular response to zinc ion | 4 | 9.91E-05 |
| BP GO:0002503 | Peptide antigen assembly with MHC class II protein complex | 3 | 2.28E-04 |
| BP GO:0045766 | Positive regulation of angiogenesis | 6 | 2.34E-04 |
| MF GO:0044548 | S100 protein binding | 3 | 0.00151091 |
| MF GO:0032395 | MHC class II receptor activity | 3 | 0.00202209 |
| MF GO:0003714 | Transcription corepressor activity | 6 | 0.00221531 |
| MF GO:0005515 | Protein binding | 53 | 0.00270962 |
| MF GO:0002020 | Protease binding | 4 | 0.0106584 |
| CC GO:0070062 | Extracellular exosome | 34 | 9.81E-08 |
| CC GO:0072562 | Blood microparticle | 8 | 6.47E-06 |
| CC GO:0009986 | Cell surface | 12 | 3.79E-05 |
| CC GO:0031902 | Late endosome membrane | 6 | 1.03E-04 |
| CC GO:0005615 | Extracellular space | 18 | 1.18E-04 |
| KEGG hsa05150 | Staphylococcus aureus infection | 7 | 5.50E-07 |
| KEGG hsa04610 | Complement and coagulation cascades | 5 | 6.45E-04 |
| KEGG hsa05310 | Asthma | 4 | 6.63E-04 |
| KEGG hsa05322 | Systemic lupus erythematosus | 6 | 9.90E-04 |
| KEGG hsa04640 | Hematopoietic cell lineage | 5 | 0.00154153 |
| *Up-regulated genes* | |  |  |
| BP GO:0007268 | Chemical synaptic transmission | 3 | 0.00293802 |
| BP GO:0007565 | Female pregnancy | 2 | 0.03138706 |
| BP GO:0070588 | Calcium ion transmembrane transport | 2 | 0.04178012 |
| MF GO:0005184 | Neuropeptide hormone activity | 2 | 0.00885525 |
| CC GO:0045202 | Synapse | 3 | 0.00143344 |
| CC GO:0043025 | Neuronal cell body | 3 | 0.0042668 |
| CC GO:0043679 | Axon terminus | 2 | 0.01570178 |
| CC GO:0005886 | Plasma membrane | 5 | 0.02634674 |

Note:If there were more than five terms enriched in this category, the top five terms were selected according to *P*-value. If there were less than five terms enriched in this category, all terms were selected. Gene count >2 and p < 0.05 were set as the threshold. DEG, differentially expressed gene; BP, biological process; CC, cellular component; MF, molecular function; GO, Gene Ontology; KEGG, Kyoto Encyclopedia of Genes and Genomes.
